# Leveraging Digital Platforms and Leadership Inclusivity to Enhance Leadership Effectiveness and Patient Outcomes in Healthcare Organizations

**DOI:** 10.3390/healthcare13151833

**Published:** 2025-07-28

**Authors:** Lina H. Khusheim

**Affiliations:** Department of Health Services and Administration, Faculty of Economics and Administration, King Abdulaziz University, Jeddah 21589, Saudi Arabia; lhkhusheim@kau.edu.sa

**Keywords:** digital platforms, inclusive leadership, healthcare outcomes, leadership effectiveness, patient satisfaction, health informatics

## Abstract

**Background:** Digital platforms and inclusive leadership are pivotal in modern healthcare, influencing organizational performance and patient outcomes. Despite the growing adoption of these factors, their combined impact on leadership effectiveness and patient care remains insufficiently understood. Prior research has primarily examined digital technology or leadership inclusivity separately, lacking integrative studies that address their joint effect on healthcare outcomes. There is a need to explore how these variables interact to improve leadership and patient-related metrics. **Methods:** This cross-sectional study surveyed 250 participants, including healthcare leaders, professionals, and patients, using structured questionnaires. The data analysis involved multiple regression, structural equation modeling (SEM), and hierarchical linear modeling (HLM) to examine the direct and hierarchical relationships among digital platform usage, leadership inclusivity, leadership effectiveness, and patient outcomes. **Results:** Leadership inclusivity showed a significant positive effect on leadership effectiveness (β = 0.16, *p* < 0.01) and patient satisfaction (β = 0.09, *p* < 0.05). Digital platform usage demonstrated a smaller but positive association with leadership effectiveness (β = 0.04) and patient satisfaction (β = 0.03). Leadership effectiveness was found to correlate moderately with patient safety (β = 0.23) and treatment efficacy (β = 0.25), with minimal organizational-level effects. **Conclusions:** This study uniquely integrates the adoption of digital technology with inclusive leadership, highlighting their synergistic influence on healthcare delivery. It advances the existing literature by providing quantitative evidence on how these elements interact to shape leadership and patient care outcomes.

## 1. Introduction

The increasing use of digital tools within healthcare is transforming how formal healthcare leaders such as department heads, unit supervisors, and hospital administrators interact with clinical teams and patient populations. For this study, ‘leaders’ are defined as individuals with decision-making authority over clinical or operational processes, including those in supervisory, managerial, or executive roles. In recent years, hospitals have utilized digital tools, including electronic health records (EHRs), telehealth, and AI, to enhance their daily tasks, interactions, and patient care [1]. Since technology plays a crucial role in healthcare, leaders must utilize it effectively to ensure better patient outcomes. Over the past few years, healthcare leaders have focused on fostering a team-based culture that incorporates input from everyone and promotes collaboration at work [2].

Leadership in healthcare is guided by digital platforms, and this should not be ignored.

Inclusive leadership enhances team openness to innovation and fosters psychological safety, which are essential for technology adoption and sustained innovation [3]. These leadership behaviors align with the core assumptions of the TAM, which emphasizes perceived usefulness and ease of use as shaped by organizational culture and support [4]. Integrating inclusive leadership with digital competency creates a participatory environment that enhances digital transformation readiness and successful implementation [5]. Appreciation and trust among team members can grow through inclusive leadership, improving their collaboration. Good performance from the team depends on having strong leadership. It has been found that when all opinions are heard and put into action at work, teams strive together, staff are happier, and fewer people quit. By encouraging everyone to join in, leaders help employees act more responsibly and honestly to protect patient safety. With more digital technology in healthcare, one must see how it influences inclusive leadership and its success. Leaders can use digital resources to check the most recent data and data analysis, which helps them decide what to do based on evidence. Leaders from these companies should lead the technological changes, keep employees informed, and ensure that everyone uses the technology together. When leaders show inclusiveness, staff are more likely to accept new changes and use digital solutions in healthcare [6]. When digital abilities and team leadership are combined, the results will likely be better for patients and team members.

While many healthcare organizations use technology widely and promote inclusion, the effect of these approaches on patients is not fully understood [7]. Because healthcare is constantly evolving and technology is improving, it is essential to consider how leadership affects the use of new digital resources. If they address this gap, researchers may learn how digital technology and inclusive practices can help leaders improve their skills and obtain better patient outcomes [8].

Building on this theoretical foundation, this study aims to examine how the integration of inclusive leadership and digital platform usage influences leadership effectiveness and patient outcomes in healthcare organizations. The existing literature underscores that participatory leadership enhances team performance and care quality, while digital tools support efficiency and timely decision-making. By analyzing these relationships jointly, this research contributes to a more nuanced understanding of how leadership behaviors and technology adoption intersect to shape organizational and clinical results.

## 2. Literature Review

The internet has introduced innovative methods to help address old problems in terms of patient care, office operations, and general health services. EHRs, telemedicine, and AI help improve communication, access to services, and decision-making in healthcare. EHR systems were made to unify patient records across health professionals. Access to healthcare is now simpler for many people, especially those far from hospitals, due to telemedicine [9,10]. Healthcare leaders use artificial intelligence (AI) to predict outcomes, allocate resources effectively, and monitor their organizations’ performance [11]. Modern technology enables organizations to operate better and allows healthcare leaders to rely on facts when making decisions. Digital platform use in this study refers to the adoption and application of technologies such as EHRs, telemedicine systems, and AI-supported clinical decision-making tools. These platforms support documentation, communication, patient monitoring, and data-driven decision-making processes.

Digital platforms and exemplary leadership in healthcare have been proven to help organizations. For example, reference [12] proposed that organizations utilizing digital innovations can expect improved decision-making and outcomes within the organization. It was observed that digital tools help manage tasks more effectively, enhance the workflow, and improve patient care. Moreover, digital technology enables leaders to adjust to various situations by presenting real-time insights and allowing them to strengthen their organizations’ ability to achieve goals [13]. Nevertheless, while digital technology in healthcare is known to be beneficial, more research is needed to understand how it can benefit leadership and patient care.

Good leadership is crucial in healthcare organizations, impacting the organization and patient care. An effective leader helps the organization achieve its goals while considering and caring for both employees and patients. In healthcare, effective leaders must possess strong communication skills, sound decision-making abilities, high levels of emotional intelligence, and the ability to motivate teams [14]. Within healthcare organizations, the best leaders help establish trust, honesty, and accountability, enabling teams to collaborate on critical tasks, especially when patient care is at stake [15].

The performance of a healthcare leader is assessed by evaluating employee satisfaction, patient care quality, and the organization’s overall functioning. Leadership effectiveness in healthcare is often assessed using scores that reflect patient satisfaction [16]. A strong leader can be recognized by staff involvement, harmonious teamwork, and sound judgment among team members. Effective leadership enables teams to work more efficiently, improve patient care, and operate more effectively. Studies have shown that hospitals where leaders focus on quality achieve better clinical results than those without this focus [17]. Leadership effectiveness in this study is conceptualized through decision-making quality, coordination capacity, communication skills, motivation of team members, and emotional intelligence, reflecting the multidimensional nature of effective leadership in healthcare.

Inclusive leadership, a key component of effective leadership in healthcare, aims to support everyone on the healthcare team through practices rooted in fairness, respect, and openness [18]. It enables leaders to value diverse perspectives, prioritize each team member’s experiences, and make decisions that benefit the entire team. Inclusive leadership fosters psychological safety and encourages participation [19], distinguishing it from transformational leadership (which focuses on vision and charisma) and transactional leadership (which emphasizes rewards and compliance). The core dimensions of inclusive leadership include openness to input, accessibility, availability for interaction, fairness of treatment, and appreciation of diversity. This study operationalizes inclusive leadership through these dimensions to ensure the construct validity when measuring its influence on leadership effectiveness and patient outcomes.

When inclusive leadership is present, teams interact more effectively, make informed decisions, and achieve better outcomes, particularly in collaboration with patients. Such leadership helps work teams build strong relationships, communicate effectively, and avoid conflicts [20]. These factors are essential for improving the workplace, enhancing employee well-being, and facilitating high performance. Inclusive leadership results in decisions that consider a wider range of factors and are therefore more practical [21]. It also ensures that healthcare teams treat all patients with cultural sensitivity, making them more satisfied and supporting better treatment outcomes, especially among diverse populations [22].

Patient outcomes measure the effectiveness of healthcare treatments in enhancing patient well-being and ensuring safety and satisfaction throughout the care process. The quality of care in any facility is largely determined by its outcomes, including patient satisfaction and perceived quality of care, as emphasized in recent regional evidence. Patient satisfaction is commonly assessed through surveys such as the HCAHPS, which evaluate provider communication, staff responsiveness, and overall patient comfort. Patient well-being is closely linked to error prevention and the overall quality of the care delivered [23]. Patient satisfaction is conceptualized as perceptions of care quality, provider communication, staff responsiveness, and overall comfort during treatment. Treatment efficacy refers to perceived health improvement, success in meeting care goals, and avoidance of errors or complications.

When leaders utilize digital tools in their leadership, patient outcomes can be significantly improved. Effective leaders who use digital technologies for care and staff communication typically have organizations with higher patient satisfaction and safety [24]. Prior studies have found that platforms like telemedicine and those utilizing AI can enhance care delivery by providing doctors with better knowledge and tools to make informed decisions [25]. Also, including more people in senior roles in healthcare helps achieve better patient results. Inclusive environments lead leaders to ensure that all members are engaged, resulting in patient care that meets the needs of diverse populations [26,27,28].

## 3. Research Methodology

To address these gaps, this study investigates the combined influence of digital platform adoption and inclusive leadership on leadership effectiveness and patient outcomes within healthcare organizations. By integrating TAM principles with inclusive leadership theory, this research offers a novel lens through which to examine the intersection of leadership behaviors and technological innovation. This study employs a multi-method statistical approach, including multiple regression, SEM, and HLM, to provide robust, multilevel insights.

### 3.1. Theoretical Relevance of the Study

This study is grounded in the intersection of the technology acceptance model (TAM) and inclusive leadership theory. The TAM posits that perceived usefulness and ease of use drive technology adoption; however, such perceptions are shaped by the leadership climate and organizational culture [29]. Inclusive leadership fosters psychological safety, participation, and trust conditions that reduce resistance to digital innovation and increase technology acceptance [20]. These leadership behaviors help employees perceive digital tools as empowering rather than disruptive. Additionally, leadership effectiveness theory explains how these behaviors directly influence team coordination, decision-making, and ultimately, patient care outcomes.

Inclusive leadership aligns with the TAM by creating enabling conditions for technology acceptance, such as verbal encouragement, shared decision-making, and psychological safety, that shape users’ perceptions of ease of use and usefulness. While the TAM is valuable for modeling acceptance behavior, it emphasizes individual-level cognition and often overlooks team dynamics, cultural factors, and contextual variance. Inclusive leadership compensates for these limitations by embedding technology use within relational, participatory, and adaptive leadership practices. Inclusive leadership moderates the relationship between digital platform characteristics and user perceptions by creating an environment where team members feel safe to provide input, learn, and experiment with technology. It mediates technology adoption by fostering a culture that views digital tools as enablers of collaboration rather than external mandates, thereby enhancing both perceived ease of use and perceived usefulness. It is conceptually distinct from transformational leadership, which emphasizes vision and charisma, and transactional leadership, which focuses on task compliance and rewards. Neither of these fully addresses the inclusive, collaborative climate required for digital innovation in healthcare. Inclusive leadership is defined in this study by five core dimensions: openness to input, accessibility, availability for interaction, fairness of treatment, and appreciation of diversity. These dimensions collectively create psychological safety, encourage participation, and support technology acceptance, offering a theoretically and contextually appropriate complement to the TAM in digitally transforming clinical environments. Together, inclusive leadership and digital platforms are theorized to jointly enhance leadership effectiveness and patient outcomes, with inclusive leadership amplifying the positive effects of digital adoption through improved team engagement and technology integration. The proposed relationships and supporting models are summarized in Table 1.

### 3.2. Causal Logic and Model Assumptions

This study posits a causal structure grounded in the TAM and leadership effectiveness theory:

1. Inclusive leadership fosters psychological safety and openness.
2. This climate enhances the acceptance and use of digital tools (the TAM’s ‘perceived usefulness’).
3. Both factors improve leadership effectiveness by enhancing decision quality and communication.
4. Leadership effectiveness then contributes to improved patient outcomes, measured through satisfaction, safety, and treatment efficacy.

This conceptual sequence justifies the directional assumptions underlying the applied analytical frameworks. Multiple regression was used to estimate the individual effects of digital platform usage and leadership inclusivity on the outcome variables, providing initial evidence of direct relationships aligned with this causal logic. SEM was employed to evaluate the complex latent constructs and the mediating relationships between the variables, testing whether leadership effectiveness serves as a pathway linking leadership and technology factors to patient outcomes as theorized. HLM accounted for the multilevel data structure and organizational nesting of participants, enabling examination of whether the strength or direction of these relationships varied across organizational contexts, thereby enriching the causal interpretation. Together, these methods form an integrated analytical strategy that incrementally tests and contextualizes the causal model across individual and organizational levels.

### 3.3. Research Design

A cross-sectional design is chosen here, as it helps investigate how digital platforms, inclusivity in leadership, and patient outcomes are associated at one moment in time. This design is used because it makes it possible to simultaneously see the present situation of leadership, tools, and health results in multiple healthcare settings. You can use correlations without wasting a lot of time following variables, making it a valuable and fast method for this study. The design can identify the connections among these variables and see their influence at a specific time, so researchers can understand the relationships and look for more places to study.

### 3.4. Sample Population and Sampling Method

This study was conducted in Saudi Arabia, focusing on healthcare institutions operating within diverse regions of the country. The research focused on healthcare organizations such as hospitals and clinics from different regions, targeting three key participant groups: healthcare leaders, healthcare professionals, and patients. These groups were selected to capture role-specific insights—leaders reported on their leadership practices, professionals assessed leadership and patient care, and patients evaluated their care experience and outcomes.

Each group was linked to specific constructs to ensure role-appropriate measurement: healthcare leaders assessed their own leadership effectiveness and inclusivity; healthcare professionals evaluated their leaders’ inclusivity and effectiveness as well as patient outcomes; and patients directly assessed patient outcomes (satisfaction, safety, treatment efficacy). This role-construct mapping ensured alignment with theoretical expectations and analytic integrity.

Participants were recruited through formal institutional coordination with selected healthcare facilities across urban and peri-urban regions, including both public and private hospitals. This regional and institutional diversity was intended to enhance the generalizability of the findings. Ethical approvals were secured from relevant oversight bodies where applicable. The inclusion criteria required that participants be active employees (for professionals and leaders) or recipients of care (for patients) within the participating organizations. The exclusion criteria included individuals who could not provide informed consent, those under 18 years of age (for patients), or submissions with incomplete survey responses.

Stratified random sampling was applied to ensure a representative and diverse sample across organizational roles and healthcare units. Stratification was performed based on participant role (leader, professional, patient), ensuring adequate representation from each group. A total of 250 participants were included, divided into three stratified clusters: healthcare leaders (*n* = 83), healthcare professionals (*n* = 84), and patients (*n* = 83). This grouping allowed for a multi-perspective comparison and supported the hierarchical nature of the study design. While this sample size supported statistical analysis using SEM and regression, subgroup analyses using HLM were interpreted cautiously due to the limited number of group-level units.

HLM was applied due to the nested structure of the data, where individuals were embedded within healthcare organizations. To justify the use of HLM, ICCs were calculated for patient safety and treatment efficacy, both exceeding the commonly accepted threshold of 0.05, indicating sufficient between-group variance. Demographic attributes such as age, gender, and education are summarized in Table 2 for each group.

### 3.5. Data Collection

Data for this study were collected through structured surveys and questionnaires, as detailed in Table 2. These instruments captured the core constructs of interest: digital platform usage, inclusive leadership, leadership effectiveness, and patient outcomes, each defined and operationalized as follows:

- Digital platform usage was measured on a 0–2 scale based on the frequency and type of digital tools used. This included specific technologies such as EHRs for unified documentation, telemedicine platforms for remote consultations, and AI-supported systems for clinical decision-making, diagnostics, and patient monitoring. These technologies support communication, data-driven decision-making, and service delivery.
- Inclusive leadership was assessed through items capturing inclusive decision-making, team empowerment, openness to diverse perspectives, fairness in leadership practices, accessibility, availability, and appreciation of diversity.
- Leadership effectiveness was evaluated using established leadership scales covering decision-making quality, coordination, motivation, communication effectiveness, and emotional intelligence. This multidimensional construct reflects a leader’s ability to guide teams, ensure collaboration, and achieve clinical and operational goals. Structured questionnaires were administered to healthcare professionals holding formal leadership roles, such as unit managers, clinical supervisors, or department heads.
- Patient outcomes were assessed across three domains—satisfaction, safety, and treatment efficacy—using validated Likert scale questions. Patient satisfaction was conceptualized through perceptions of care quality, communication with providers, staff responsiveness, and overall comfort during treatment. Treatment efficacy included perceived health improvement, success in meeting care goals, and avoidance of errors or complications, reflecting both subjective and process-based outcomes.

The data linkage was explicitly structured so that leaders reported on self-perceived leadership behaviors; professionals provided external evaluations of leadership and patient outcomes within their units; and patients reported directly on their care experience. This design enabled multi-perspective validation of constructs and supported the multilevel structure applied in HLM.

All the instruments used in this study were adapted from previously validated scales in the leadership, healthcare quality, and technology acceptance literature. Sources included scales such as the Inclusive Leadership Scale by [11], leadership effectiveness metrics adapted from Yukl’s Leadership Behavior Inventory, and technology use measures based on Davis’s TAM framework. A pilot study was conducted to ensure contextual relevance, followed by a psychometric evaluation of the survey tools. The internal consistency for each construct was assessed using Cronbach’s alpha, with all the values exceeding the acceptable threshold of 0.70. Specifically, Cronbach’s alpha was 0.81 for inclusive leadership, 0.84 for leadership effectiveness, 0.76 for digital platform usage, and 0.79 for patient outcomes, indicating acceptable to high reliability across constructs. Additionally, exploratory factor analysis (EFA) was used to confirm the dimensional structure and item loadings, and confirmatory factor analysis (CFA) was applied to validate the overall model fit before SEM. The CFA results showed acceptable fit indices (e.g., CFI = 0.92, RMSEA = 0.06), supporting the construct validity. These procedures established the reliability and construct validity of the measurement instruments.

Surveys and questionnaires help collect data from a large group of people in a planned and verified way. As a result, research can identify connections between effective leadership, using technology, and patient health. All the questionnaire items were adapted from previously validated instruments in the leadership, healthcare quality, and technology acceptance literature. Minor contextual adjustments were pilot-tested before distribution. A psychometric evaluation was conducted to ensure reliability and construct validity.

### 3.6. Data Analysis

This study employed a multi-method analytical strategy, where each technique served a distinct purpose aligned with theoretical and structural considerations. This layered approach was designed to provide both breadth and depth in examining the relationships among digital platform usage, inclusive leadership, leadership effectiveness, and patient outcomes, ensuring coherence across levels of analysis. Multiple regression was used for the initial hypothesis testing and to examine the individual effects of digital platform usage and inclusive leadership on leadership effectiveness and patient outcomes, allowing the identification of the unique contribution of each predictor variable while controlling for others. These analyses served as a foundation for subsequent modeling, offering baseline estimates of direct effects and clarifying variable relationships at the observed level.

SEM was then applied to explore the more complex, mediating relationships between latent constructs and to assess the overall model fit based on the TAM and leadership theory, being particularly useful for modeling indirect paths and validating construct interrelationships. SEM extended the regression findings by incorporating latent variables, simultaneously testing direct and indirect effects (e.g., the mediating role of leadership effectiveness between inclusive leadership/digital platform usage and patient outcomes). This integration allowed us to confirm whether the observed patterns in the regression models held when the measurement error was accounted for and the theoretical constructs were modeled explicitly. Finally, HLM was used to account for the nested structure of the data, incorporating variance at the organizational (group) level, which allowed analysis of cross-level interactions and group-level effects such as organizational digital maturity or leadership climate. HLM complemented SEM by testing whether organizational-level variance influenced the strength or direction of the relationships identified in the individual-level analyses, thereby aligning the micro- and macro-level insights. This approach ensured that the results across analytical methods were cumulative rather than fragmented, with each method building upon and contextualizing the findings of the prior. Together, these methods provided a coherent analytic structure: regression established baseline associations; SEM tested latent constructs and mediating relationships; and HLM explored cross-level variance, allowing an integrated understanding of how leadership and technology variables jointly shape outcomes.

While SEM modeled the mediating role of leadership effectiveness between inclusive leadership, digital platform use, and patient outcomes, HLM further contextualized these relationships by revealing whether they were consistent across organizational units. Future research could formally test the theorized moderating role of inclusive leadership in the relationship between digital platform characteristics and perceived ease of use or usefulness. This would further clarify the causal mechanisms by which inclusive leadership shapes technology adoption processes within healthcare organizations.

#### Pearson Correlation Coefficient

The Pearson correlation coefficient was used to measure the strength and direction of the linear associations between variables:

$$r=\;\frac{\sum \left(xi-\bar{x})(yi-\bar{y}\right)}{\sqrt{\sum (}xi-\bar{x}{)}^{2}\;\sum (yi-\bar{y}{)}^{2}}$$

where:

- r = correlation coefficient
- xi, yi = observed values of variables X and Y
- $\bar{x}$, $\bar{y}$ = means of X and Y, respectively

This formula evaluates the linear relationship between two continuous variables. In the context of this study, it measures the strength and direction of associations, such as those between inclusive leadership and patient satisfaction or between digital tool use and leadership effectiveness.

Next, multiple regression analysis assessed the unique contribution of digital platform usage and leadership inclusivity to leadership effectiveness and patient outcomes. This technique helps identify which factors have the most decisive influence on these while controlling other variables.

Y=β0+β1X1+β2X2+ε

where:

Y = dependent variable (e.g., leadership effectiveness or patient satisfaction)
X1, X2 = independent variables (e.g., digital platform usage and inclusive leadership)
β0 = intercept
β1, β2 = regression coefficients
ε = error term

SEM was applied to explore these relationships further and more complexly. SEM allows for testing multiple relationships simultaneously and provides a more detailed understanding of how leadership inclusivity and digital platforms interact to influence the outcomes between the latent and observed variables:

η=Bη+Γξ+ζ

where:

η is the vector of the endogenous latent variables (e.g., leadership effectiveness)
ξ is the vector of the exogenous latent variables (e.g., digital platform usage)
B and Γ are coefficient matrices
ζ is the vector of structural disturbances

It shows how leadership inclusivity, the use of digital platforms, and how well leaders perform are connected to patient outcomes, strengthening the connections between them. HLM was also be employed because the data includes multiple tiers, such as groups composed of different participants (healthcare leaders, staff, and patients). HLM uncovers the unique roles digital platforms and inclusive leadership have at the individual, team, and organizational levels. In the HLM, participants were nested within healthcare organizations, with professionals and patients grouped under their corresponding leadership units. Leaders formed the Level 2 grouping variable, reflecting organizational and leadership climate influences on individual-level outcomes. This nesting logic aligns the analytic framework with the role-specific data collection structure.

Level 1 (individual-level model):

Yij=β0j+β1jXij+rij

Level 2 (group-level model):

β0j=γ00+γ01Wj+u0j

In this framework:

- Yij is the outcome for individual i in group j
- Xij is the individual-level predictor (e.g., perceived inclusivity)
- Wj is the group-level predictor (e.g., organizational digital maturity)
- γ00, γ01 are fixed effects
- u0j and rij are the random effects at the group and individual levels

These combined statistical approaches ensure a comprehensive and multi-layered understanding of how leadership inclusivity and digital platform use affect leadership effectiveness and patient outcomes. The path structure was guided by the TAM and inclusive leadership theory, which posit that digital tool adoption and leadership behaviors shape team processes and outcomes through leadership effectiveness as a mediating construct. The SEM model was evaluated using standard fit indices: comparative fit index (CFI = 0.934), root mean square error of approximation (RMSEA = 0.058), and standardized root mean square residual (SRMR = 0.049)—all within accepted thresholds, confirming a satisfactory model fit. Inclusive leadership was modeled as a latent construct comprising openness to input, accessibility, availability for interaction, fairness of treatment, and appreciation of diversity, with these dimensions measured through the corresponding indicators in the SEM framework. This explicit modeling of inclusive leadership ensures construct validity and aligns the analysis with the theoretical framework proposed in this study.

**Figure 1:**
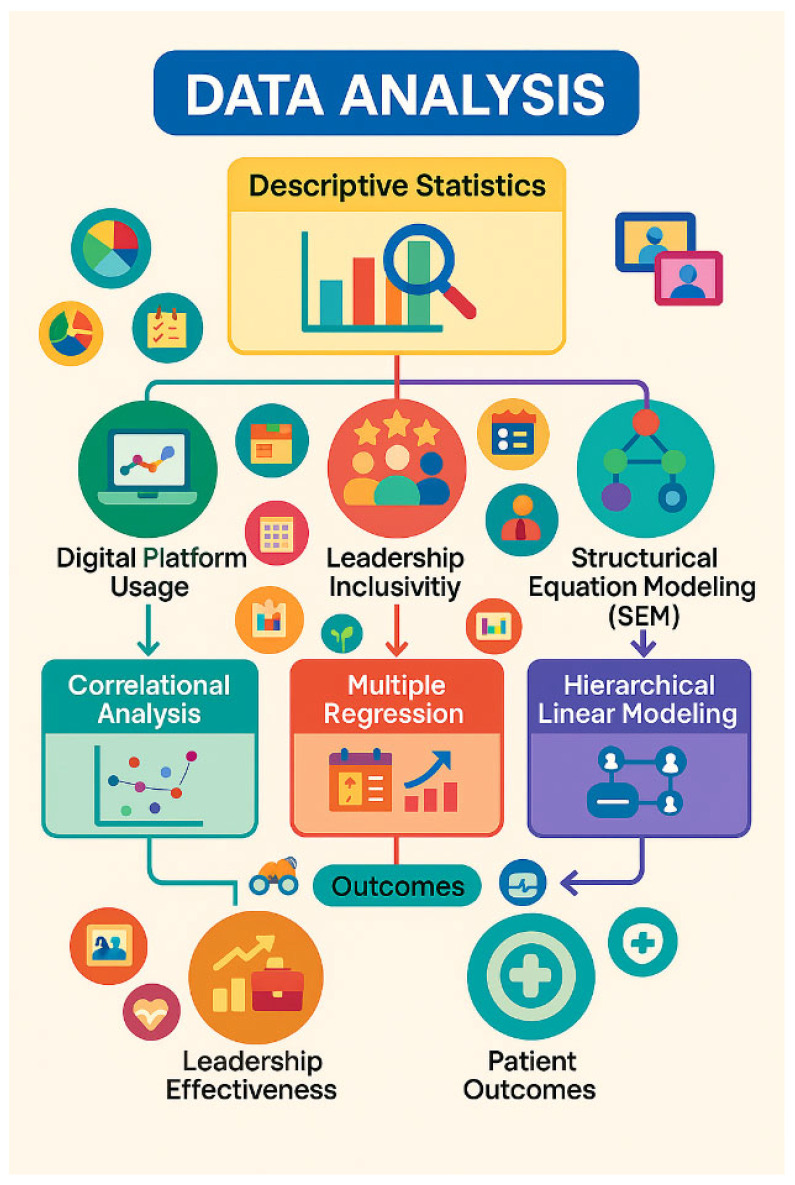
Analytical framework of the study.

To assess the connections between leadership, digital tools, and patient outcomes, diverse analytic methods are needed, and you can see this process in Figure 1. Correlational analysis and multiple regression will allow you to see the relationships between your variables. Aside from that, SEM and HLM will help understand the reasons for different relationships and differences between groups. Applying various techniques will show that the analysis is complete and can uncover understandable and surprising results. They will be the starting point for creating helpful suggestions regarding how healthcare organizations can improve leadership and use technology for patient care. The SEM model was assessed using standard fit indices: comparative fit index (CFI = 0.934), root mean square error of approximation (RMSEA = 0.058), and standardized root mean square residual (SRMR = 0.049), all indicating an acceptable model fit based on established thresholds. The model structure was developed based on theoretical assumptions where leadership inclusivity and digital platform usage serve as exogenous predictors influencing leadership effectiveness, which mediates patient outcomes. Leadership inclusivity was modeled as a latent construct consisting of openness to input, accessibility, availability for interaction, fairness of treatment, and appreciation of diversity, measured through the corresponding indicators in the SEM framework to ensure alignment with this study’s theoretical foundation.

## 4. Results

The results are presented following this study’s multi-method analytic structure to ensure clarity and coherence across techniques. First, multiple regression analysis provides baseline evidence of the direct effects of digital platform usage and inclusive leadership on leadership effectiveness and patient outcomes. Next, SEM deepens this analysis by modeling latent variables and testing mediating pathways, particularly the role of leadership effectiveness in linking inclusive leadership and digital platform usage to patient outcomes. Finally, HLM captures cross-level and organizational effects, addressing group-level variance in outcomes such as patient safety and treatment efficacy. This integrated analytic sequence ensures that each method builds upon the findings of the previous, contributing to a coherent and cumulative argument about the interplay between leadership, technology adoption, and patient care.

Table 3 presents descriptive statistics for key variables in this study, including digital platform usage, leadership inclusiveness, leadership effectiveness, and patient outcomes. The mean values for digital platform usage and leadership inclusivity suggest moderate adoption of digital tools and inclusive leadership practices, with digital platform usage being slightly lower (mean = 0.97). Leadership effectiveness shows a moderate score (mean = 5.90), with patient satisfaction, safety, and treatment efficacy also demonstrating high mean scores (around 7). The age group mean indicates a younger population (mean = 21.84). The significance of these findings lies in understanding the relationships between leadership inclusivity, digital platform usage, and patient outcomes. The moderate scores for digital platform usage and leadership inclusivity suggest an opportunity for improvement in integrating these practices into healthcare organizations, as higher inclusivity and practical digital tool usage are correlated with better patient care.

The correlation matrix in Table 4 highlights key relationships between the study variables, offering insight into both expected and unexpected patterns. While digital platform usage shows a negligible association with leadership effectiveness (r = –0.00827), this weak relationship underscores that technology alone may not translate into leadership improvement without a supportive behavioral and organizational context. Leadership inclusivity exhibits a small positive correlation with leadership effectiveness (r = 0.1612), reinforcing the view that inclusive leadership contributes to better team coordination and engagement. Notably, leadership effectiveness is moderately correlated with patient safety (r = 0.2295) and treatment efficacy (r = 0.2515), suggesting that leadership quality significantly affects how safely and effectively care is delivered. However, the relatively weak effects of both digital platform usage and leadership inclusivity on treatment efficacy may reflect external influences, such as clinical expertise, resource constraints, or patient conditions, that go beyond leadership control.

**Figure 2:**
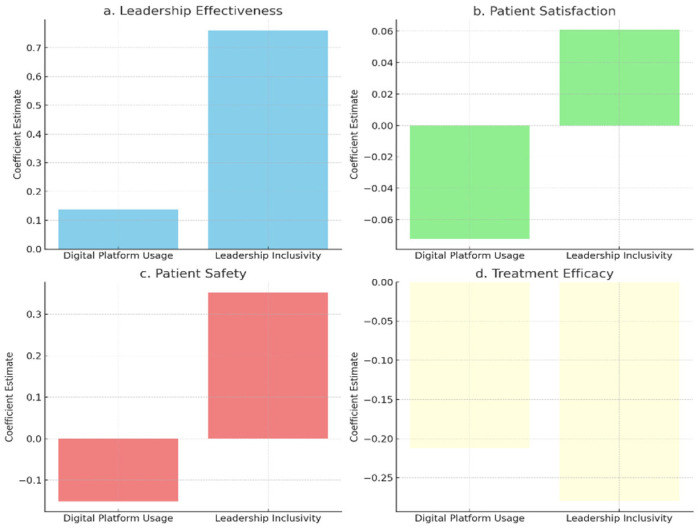
Impact of leadership effectiveness on patient safety.

Figure 2 shows that leadership inclusivity has a more substantial positive impact on leadership effectiveness and patient safety than digital platform usage, highlighting the importance of inclusive leadership in enhancing these outcomes. Both variables positively influence patient satisfaction, but the effect is weaker for digital tools, indicating that leadership quality plays a more significant role in shaping patient experiences. Treatment efficacy shows minimal influence from both leadership inclusivity and digital platform usage. This raises important considerations: treatment outcomes often depend on clinical protocols, staff expertise, and medical infrastructure—areas that may not be significantly affected by leadership style or digital systems alone. This aligns with critiques of the TAM, which focuses on individual-level adoption and may not fully account for the complex clinical drivers of efficacy in healthcare settings.

**Figure 3:**
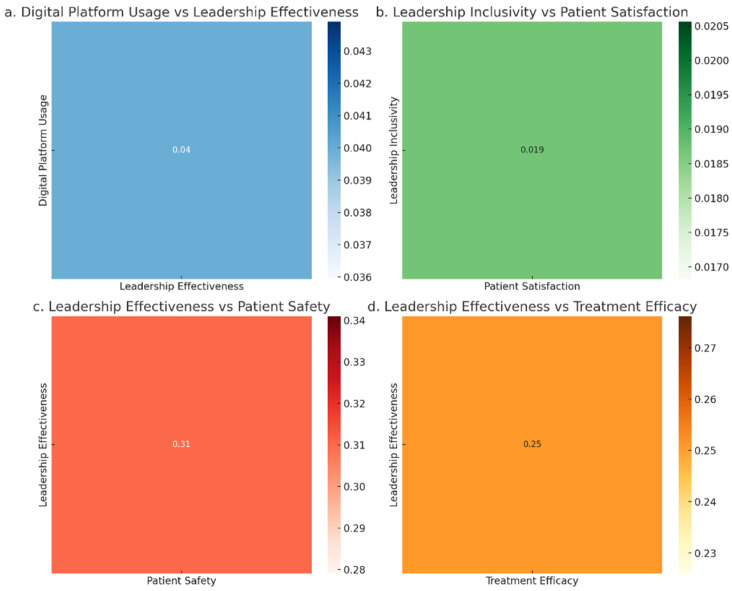
Relationship between digital platform usage, leadership inclusivity, effectiveness, and patient outcomes.

Figure 3 shows the relationships between digital platform usage, leadership inclusivity, leadership effectiveness, and patient outcomes (satisfaction, safety, and treatment efficacy). Digital platform usage exhibits a very weak correlation with leadership effectiveness (r = 0.04), indicating that digital tools alone have a minimal impact on leadership outcomes. Leadership inclusivity shows a small positive effect on patient satisfaction (r = 0.02), suggesting that inclusivity contributes to better patient experiences. Leadership effectiveness has a moderate positive impact on patient safety (r = 0.31) and treatment efficacy (r = 0.25), highlighting its critical role in improving patient care. Digital platforms, while beneficial, appear less impactful in driving these outcomes compared to leadership practices.

**Figure 4:**
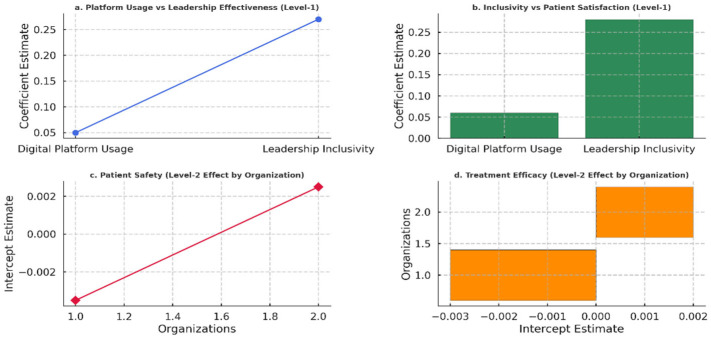
Impact of leadership inclusivity and digital platform usage on healthcare outcomes.

Figure 4 illustrates the relationships between digital platform usage, leadership inclusivity, and key outcomes, including leadership effectiveness, patient satisfaction, patient safety, and treatment efficacy. The findings show that leadership inclusivity substantially influences leadership effectiveness and patient satisfaction more than digital platform usage, which has a relatively weaker but positive effect. The random interceptions for patient safety and treatment efficacy indicate minimal variability across organizations at the group level.

## 5. Discussion

This study examined how digital platform usage and inclusive leadership jointly influence leadership effectiveness and patient outcomes in Saudi healthcare settings. The findings contribute to the growing body of literature linking leadership dynamics with technology adoption in achieving healthcare quality targets aligned with Vision 2030.

Consistent with earlier studies, inclusive leadership showed a moderate positive association with leadership effectiveness and patient satisfaction, aligning with research that emphasizes the role of participatory, emotionally intelligent leadership in healthcare outcomes [21,22]. Prior work suggests that leaders who promote inclusivity foster trust, shared decision-making, and team cohesion, which are essential for high-performing healthcare teams [26]. These results reinforce the importance of psychological safety and verbal encouragement as mediators of leadership influence in healthcare environments.

Leadership effectiveness, in turn, demonstrated significant correlations with patient safety and treatment efficacy, which is in line with studies that associate effective leadership with fewer medical errors, stronger communication, and improved clinical outcomes [16,17,20]. These findings indicate that leadership is not only a managerial function but also a critical determinant of healthcare delivery quality. While digital platforms such as EHRs and telemedicine tools had a weaker but positive relationship with leadership effectiveness and patient satisfaction, their influence on treatment efficacy was limited—echoing critiques of the TAM that highlight its focus on individual-level perception and lack of contextual sensitivity in complex clinical environments [4,5,25].

The findings of this study highlight limited direct effects of digital platforms on patient outcomes. Prior research, such as [12], has documented stronger digitalization impacts in organizational settings. This contrast may reflect contextual differences, such as the stage of digital transformation across Saudi healthcare institutions. The maturity and integration of digital tools may not yet be sufficient to yield more substantial outcome effects.

Additionally, several unmeasured confounders may have influenced the observed relationships. Factors such as organizational size, availability of clinical or technological resources, and presence of structured leadership development programs could moderate the effectiveness of inclusive leadership and digital adoption. For instance, larger or better-resourced hospitals may have more robust digital infrastructures and established leadership training pathways, which could enhance the practical impact of both technology and leadership on patient outcomes. Future research should consider incorporating these contextual moderators to better isolate the true effects of digital and leadership variables.

The results suggest that while technology acts as an enabler, its transformative potential is realized only when paired with strong, inclusive leadership. This underscores the need for integrated interventions that promote both digital capacity and leadership development. Importantly, the random intercepts for patient safety and treatment efficacy at the organizational level suggest that context still matters, indicating that broader systemic and infrastructural factors may moderate the outcome variability.

Importantly, the combined use of multiple regression, SEM, and HLM strengthens this study’s conclusions. Each method provided complementary insights—direct effects (regression), mediating relationships (SEM), and organizational context (HLM)—that collectively offer a more comprehensive understanding of how inclusive leadership and digital platforms interact to shape leadership effectiveness and patient outcomes. This integrated analytic approach enhances confidence in the findings and highlights the multi-layered nature of leadership and technology effects in healthcare.

The practical implications of these findings are substantial. Healthcare institutions should invest in inclusive leadership training programs that emphasize empathy, team empowerment, and collaborative decision-making. Simultaneously, the deployment of digital tools should be guided by leadership capable of facilitating technology integration through communication, role modeling, and support systems. Such alignment can help ensure that technological innovation translates into meaningful clinical and operational improvements.

Despite its contributions, this study has limitations. Its cross-sectional design restricts causal inference, and the self-reported nature of the survey may introduce response bias. While the stratified sampling ensures diversity, the sample size may limit generalizability to all healthcare systems across the country. Additionally, this study focused primarily on perceptual and leadership-level indicators without integrating clinical or operational performance metrics.

Future research could benefit from using longitudinal or experimental designs to assess causality and change over time. Further exploration of specific digital innovations (e.g., AI-based decision support, virtual care platforms) and their alignment with leadership styles may provide deeper insights. Future studies should also formally examine the theorized moderating role of inclusive leadership in the relationship between digital platform characteristics and perceived ease of use or usefulness, aiming to clarify the causal mechanisms by which leadership shapes technology adoption. Moreover, investigating how organizational culture, resource availability, or team structure mediate the effectiveness of inclusive leadership could inform more context-sensitive models of healthcare improvement.

## 6. Policy Framework

The findings of this study suggest several important policy implications for healthcare organizations, including improving leadership effectiveness and patient care.

1. Promoting inclusive leadership: Healthcare organizations should prioritize diversity in leadership teams. Policies should emphasize inclusivity to enhance team dynamics and patient outcomes. Action steps include diversity training for leadership and evaluating inclusivity through surveys.
2. Leveraging digital platforms as enablers: Digital tools support leadership effectiveness and patient care when integrated with inclusive practices. Policies should promote targeted use of platforms like EHRs and telemedicine to complement leadership efforts, supported by ongoing staff training. Given that this study found only modest direct effects of digital platform usage on patient outcomes, policies should emphasize that technology investments alone are insufficient. Digital platform deployment should be paired with leadership development and culture-building initiatives to ensure meaningful and sustained improvements in patient care.
3. Leadership development programs: Investment in leadership development is key. Programs should focus on inclusive leadership practices and the effective utilization of digital tools. Mentorship and performance metrics can help track leadership effectiveness.
4. Enhancing patient-centered care: Inclusive leadership has improved patient satisfaction. Policies should promote patient-centered care strategies that involve patients in decision-making processes. This includes gathering regular patient feedback and training healthcare professionals in effective patient engagement.
5. Monitoring and evaluation: Regular leadership and digital platform usage assessment should be mandated. Both leadership effectiveness and platform integration should be part of the organizational KPIs, ensuring continuous improvement.

**Figure 5:**
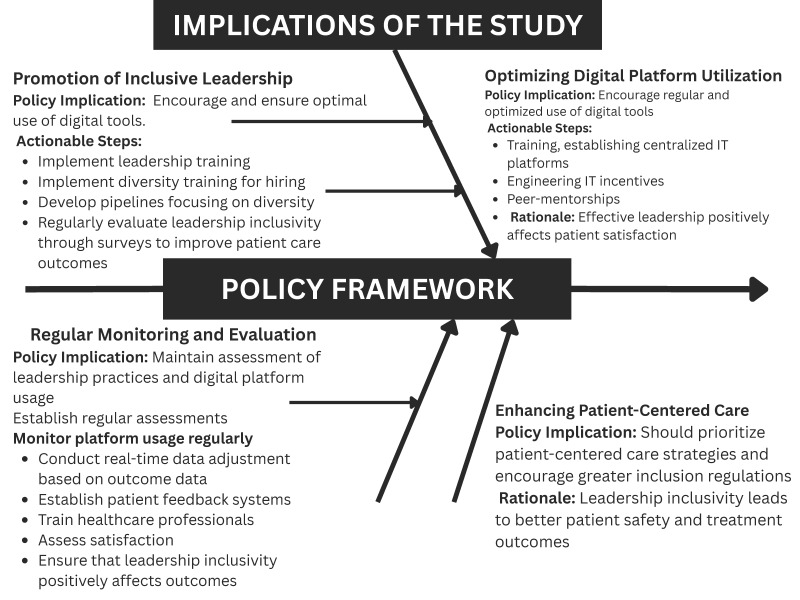
Policy framework for healthcare organizations.

The policies in Figure 5 align with the existing literature, which emphasizes the role of inclusive leadership in improving outcomes and the supportive role of technology [11,28]. Healthcare organizations can effectively improve leadership practices and patient care outcomes by adopting this framework.

## 7. Conclusions

This study reveals that leadership inclusivity significantly enhances leadership effectiveness and patient satisfaction, while digital platform usage plays a supportive role, contributing positively when integrated with strong leadership practices. Leadership effectiveness is further associated with improved patient safety and treatment efficacy, indicating that inclusive leadership practices and effective digital engagement collectively support high-quality care delivery. These results underscore the central role of inclusive leadership in driving better healthcare experiences and outcomes, with digital tools providing meaningful support to leadership functions. These findings suggest that while digital platforms provide valuable infrastructure, their direct effects on patient outcomes may be limited unless combined with strong inclusive leadership that fosters team engagement, trust, and effective use of technology. Therefore, recommendations for digital system implementation should emphasize integration with leadership development and organizational culture initiatives rather than standalone adoption. This study’s limitations include its cross-sectional design, which limits the ability to establish causal relationships. Additionally, data was collected at a single point in time, which may not capture long-term trends. The sample size, while sufficient, may not be fully representative of all healthcare settings, and the study focuses primarily on self-reported data, which could introduce bias. The analysis did not formally assess the mediation pathways, which limits understanding of whether the effects of digital platforms operate indirectly through leadership factors. Future research should incorporate mediation models to better evaluate these mechanisms. Additionally, the observed association between leadership inclusivity and patient satisfaction may reflect bidirectional influences, where higher satisfaction could influence perceptions of leadership. Longitudinal or experimental designs are recommended to clarify the directionality of these relationships. Future studies could also explore the role of specific digital tools, such as AI and telemedicine, in different healthcare environments. Further examination of how leadership inclusivity interacts with contextual factors like organizational culture and resource availability would enrich the understanding. Expanding the research to diverse healthcare systems globally would enhance the generalizability of the findings.

## Figures and Tables

**Table 1:** Theoretical relevance of this study.

Theory	Core Concepts from the Literature	Study Variables Mapped	Subdimensions/Components	Operationalization/Measurement	Analytical Techniques Used
TAM	Perceived usefulness, perceived ease of use, and organizational support	Digital platform usage (type, frequency), influenced by leadership climate	EHRs, telemedicine, AI tools; user perceptions of usefulness and ease of use	0–2 scale measuring frequency and scope of use + survey items on perceived usefulness/ease of use	Correlational analysis, SEM
Inclusive Leadership Theory	Psychological safety, trust, team inclusion, and participatory decision-making	Inclusive leadership (diversity in input, openness, fairness)	Openness to input, accessibility, availability for interaction, fairness, diversity appreciation	Latent construct in SEM, measured via survey items adapted from validated inclusive leadership scales	Structured questionnaires, multiple regression
Leadership Effectiveness Theory	Motivation, decision-making, coordination, and influence on performance	Leadership effectiveness (measured via scale), patient outcomes (safety, satisfaction, efficacy)	Decision-making, communication, coordination, motivation, emotional intelligence	Leadership effectiveness scale, latent in SEM; patient outcomes via Likert scale items	Multiple regression, SEM, HLM

**Table 2:** Participants’ data and construct specification.

Participant Group	Variables Measured	Measurement Method	Participants’ Data (from the Dataset)	Psychometric Properties	Subdimensions/Components
Healthcare Leaders and Managers	Leadership Effectiveness, Leadership Inclusivity	Structured questionnaires assessing leadership effectiveness and inclusivity (e.g., diversity in teams, decision-making, team engagement)	Age Group (21.84), Gender (57% Female, 43% Male), Education Level (60% Bachelor’s, 30% Master’s)	Cronbach’s alpha > 0.70; EFA confirmed structure; CFA validated model fit	Leadership Effectiveness: decision-making, coordination, communication, motivation, emotional intelligence Inclusive Leadership: openness to input, accessibility, availability, fairness, diversity
Healthcare Professionals (Doctors, Nurses, Admin)	Leadership Effectiveness, Leadership Inclusivity, Patient Outcomes	Structured questionnaires assessing perceptions of leadership, inclusivity, and patient outcomes (e.g., satisfaction, safety)	Age Group (21.84), Role in Organization (40% Doctors, 40% Nurses, 20% Admin)	Cronbach’s alpha > 0.70; EFA confirmed structure; CFA validated model fit	Patient Outcomes: satisfaction (communication, responsiveness, comfort), safety, treatment efficacy (health improvement, goal achievement, error avoidance)
Patients	Patient Outcomes (Satisfaction, Safety, Treatment Efficacy, Perceived Quality of Care)	Surveys measuring satisfaction, safety, treatment efficacy, and quality of care	Age Group (Varies across 18–30), Gender (Based on patient group responses)	Cronbach’s alpha > 0.70; EFA confirmed structure; CFA validated model fit	Satisfaction: care quality, communication, staff responsiveness, comfort Treatment Efficacy: health improvement, goal attainment, error avoidance
Digital Platform Usage	Digital Platform Usage (frequency, type, scope)	A separate set of questions assessing the usage of platforms like EHR, telemedicine, and AI tools	Digital Platform Usage (0–2 scale; 0 = Low, 1 = Medium, 2 = High)	Cronbach’s alpha > 0.70; EFA confirmed structure; CFA validated model fit	EHR, telemedicine, AI-supported decision tools

**Table 3:** Summary statistics of the study’s key variables.

Variable	Mean	Std	Min	25%	50%	75%	Max
Digital Platform Usage	0.972	0.793558	0	0	1	2	2
Leadership Inclusivity	1.032	0.71626	0	1	1	2	2
Leadership Effectiveness	5.897583	2.601424	1.570375	3.60021	5.764921	7.840718	10.87864
Patient Satisfaction	6.939238	2.360504	1.942683	4.830841	7.053726	9.145321	10
Patient Safety	7.344414	2.482879	1.811206	5.160761	7.932022	10	10
Treatment Efficacy	7.06299	2.481617	1.744596	5.073105	7.370792	9.471482	10
Age Group	21.84	3.940114	18	18	22	26	30

**Table 4:** Interrelationship between the key study variables.

Variable	Digital Platform Usage	Leadership Inclusivity	Leadership Effectiveness	Patient Satisfaction	Patient Safety	Treatment Efficacy	Age Group
Digital Platform Usage	1	−0.04081	−0.00827	0.076201	0.089329	−0.0224	−0.00658
Leadership Inclusivity	−0.04081	1	0.161243	0.09356	0.026528	0.081806	−0.00387
Leadership Effectiveness	−0.00827	0.161243	1	0.095479	0.229504	0.251512	−0.04288
Patient Satisfaction	0.076201	0.09356	0.095479	1	−0.00417	−0.01201	0.073831
Patient Safety	0.089329	0.026528	0.229504	−0.00417	1	0.081117	−0.03178
Treatment Efficacy	−0.0224	0.081806	0.251512	−0.01201	0.081117	1	−0.04204
Age Group	−0.00658	−0.00387	−0.04288	0.073831	−0.03178	−0.04204	1

## Data Availability

The data that support the findings of this study are available on request from the corresponding author.
